# Influence of companion diagnostics on efficacy and safety of targeted anti-cancer drugs: systematic review and meta-analyses

**DOI:** 10.18632/oncotarget.5946

**Published:** 2015-10-04

**Authors:** Alberto Ocana, Josee-Lyne Ethier, Laura Díez-González, Verónica Corrales-Sánchez, Amirrtha Srikanthan, María J. Gascón-Escribano, Arnoud J. Templeton, Francisco Vera-Badillo, Bostjan Seruga, Saroj Niraula, Atanasio Pandiella, Eitan Amir

**Affiliations:** ^1^ Medical Oncology Department, Albacete University Hospital and Translational Research Unit, Albacete, Spain; ^2^ Division of Medical Oncology and Hematology, Princess Margaret Cancer Centre and the Department of Medicine, University of Toronto, Toronto, Canada; ^3^ Department of Medical Oncology and Hematology, Kantonsspital St. Gallen, Switzerland; ^4^ Department of Medical Oncology, Institute of Oncology, Ljubljana, Slovenia; ^5^ Department of Medical Oncology and Hematology, CancerCare Manitoba and University of Manitoba, Winnipeg, Canada; ^6^ Centro de Investigación del Cáncer CIC-CSIC, Universidad de Salamanca, Salamanca, Spain

**Keywords:** cancer drugs, companion diagnostics, efficacy, trial design

## Abstract

**Background:**

Companion diagnostics aim to identify patients that will respond to targeted therapies, therefore increasing the clinical efficacy of such drugs. Less is known about their influence on safety and tolerability of targeted anti-cancer agents.

**Methods and findings:**

Randomized trials evaluating targeted agents for solid tumors approved by the US Food and Drug Administration since year 2000 were assessed. Odds ratios (OR) and and 95% confidence intervals (CI) were computed for treatment-related death, treatment-discontinuation related to toxicity and occurrence of any grade 3/4 adverse events (AEs). The 12 most commonly reported individual AEs were also explored. ORs were pooled in a meta-analysis. Analysis comprised 41 trials evaluating 28 targeted agents. Seventeen trials (41%) utilized companion diagnostics. Compared to control groups, targeted drugs in experimental arms were associated with increased odds of treatment discontinuation, grade 3/4 AEs, and toxic death irrespective of whether they utilized companion diagnostics or not. Compared to drugs without available companion diagnostics, agents with companion diagnostics had a lower magnitude of increased odds of treatment discontinuation (OR = 1.12 versus 1.65, *p* < 0.001) and grade 3/4 AEs (OR = 1.09 versus 2.10, *p* < 0.001), but no difference in risk of toxic death (OR = 1.40 versus 1.27, *p* = 0.69). Differences between agents with and without companion diagnostics were greatest for diarrhea (OR = 1.29 *vs*. 2.43, *p* < 0.001), vomiting (OR = 0.86 *vs*. 1.44, *p* = 0.005), cutaneous toxicity (OR = 1.82 *vs*. 3.88, *p* < 0.001) and neuropathy (OR = 0.64 *vs*. 1.60, *p* < 0.001).

**Conclusions:**

Targeted drugs with companion diagnostics are associated with improved safety, and tolerability. Differences were most marked for gastrointestinal, cutaneous and neurological toxicity.

## INTRODUCTION

The aim of personalizing cancer treatments by targeting genomic alterations in the tumor has seen rapid advances in recent years [1]. Knowledge of the molecular mechanisms associated with cancer initiation and progression has recently improved with the increasing use of molecular techniques such as gene sequencing [1, 2]. This knowledge has permitted the identification of alterations in cancer cells and the subsequent development of therapeutic agents targeting these alterations [3, 4]. Central to the drive for personalized medicine is the need to develop and validate specific diagnostic tests that facilitate the identification of patients that are most likely to respond to a given treatment [5]. Such tests have been named companion diagnostics and are defined by the US Food and Drug Administration (FDA) as *in vitro* diagnostic devices that provide information that is essential for the safe and effective use of a corresponding therapeutic product [6]. A companion diagnostic is a pre-treatment predictor of benefit that is expected to allow selection of patients with a biomarker predicting greater efficacy from a given therapy compared to patients without the biomarker treated with the same drug. In parallel with the development of companion diagnostics, the identification of tumors where specific molecular alterations are ubiquitous has also permitted the design and evaluation of agents targeting these molecular alterations [4]. This has been the case for agents targeting *c-kit* in gastrointestinal stromal tumors (GIST) [7-8] or *RET* in medullary thyroid cancer [9, 10].

Some companion diagnostics have been developed using retrospective analyses of clinical studies [11-12] while others have been developed synchronously with the related experimental drug and have been incorporated in multiples steps of the drug development process, including randomized controlled trials (RCTs) [5]. Presently, there are few targeted drugs in clinical development that do not have an associated biomarker discovery program to identify a specific companion diagnostic [5]. Despite these advances, little is known about the influence of the availability of companion diagnostics on safety and tolerability of targeted agents.

Here, we aimed to evaluate the impact of incorporating companion diagnostics in randomized controlled trials (RCTs) of targeted agents that were used for the approval of new drugs since 2000. We hypothesized that the identification of responding patients by using companion diagnostics would be associated with not only increased magnitude of clinical benefit but also reduced toxicity compared to targeted agents without accompanying diagnostic tests.

## RESULTS

**Figure 1:**
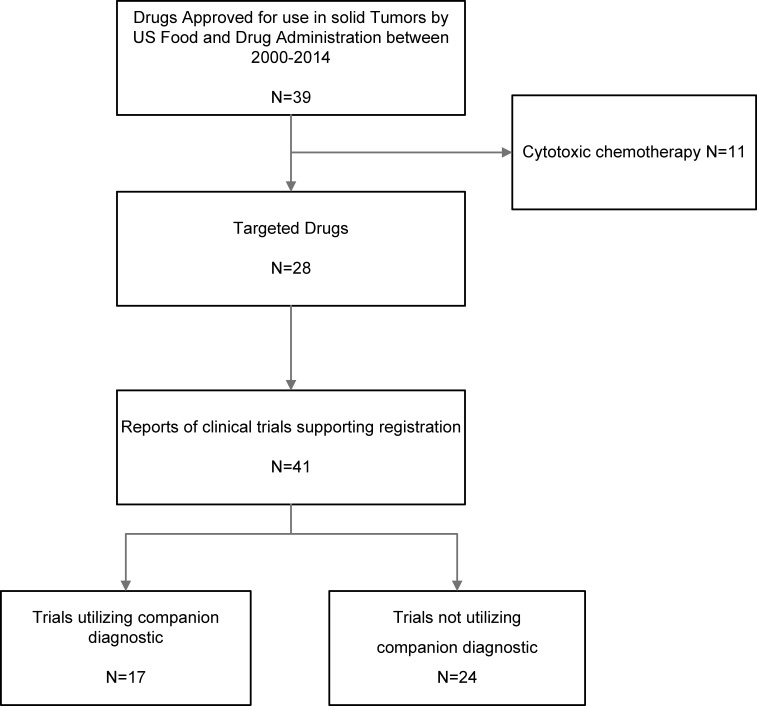
Flow diagram for identification and inclusion of studies

We identified 28 different targeted anti-cancer agents approved by the US FDA for 15 different indications between January 2000 and April 2014. These agents were evaluated in 41 separate trials. Seventeen trials (41%) were categorized as having a companion diagnostic (including 4 studies in tumors with ubiquitous molecular alterations, see Figure 1). Characteristics of included studies are shown in Table 1 and a list of drugs, their licensed indications and subgroup category is shown in Supplementary Table 1. Studies of drugs with companion diagnostics generally had lower sample sizes (Mann-Whitney U *p* = 0.03) and were more likely to use PFS rather than OS as the primary endpoint (Mann-Whitney U *p* = 0.01). A non-significantly higher proportion of studies of targeted drugs with companion diagnostics led to accelerated approval by the FDA compared with regular approval (Mann-Whitney U *p* = 0.18). A list of FDA approved diagnostic tests identified in our search is shown in Supplementary Table 2.

**Table 1:** Characteristics of included studies

Characteristic	Companion Diagnostics (n=17)	Non-companion Diagnostics (n=24)
Sample size Median Range	347 199-1217	637 171-1226
Disease Site Breast Cancer Colorectal Cancer Non-small cell lung cancer Melanoma Renal cell carcinoma Medullary thyroid cancer Gastrointestinal Stomal Tumor Other	4 (24%) 2 (12%) 3 (18%) 3 (18%) 0 (0%) 2 (12%) 2 (12%) 1 (6%)	2 (8%) 7 (29%) 2 (8%) 0 (0%) 6 (25%) 0 (0%) 0 (0%) 7 (29%)
Primary Endpoint Overall survival Progression-free survival or time to progression	3 (18%) 14 (82%)	14 (58%) 10 (42%)
Cross-over	6 (35%)	6 (25%)
Quality of life reported	4 (24%)	13 (54%)
Type of FDA approval Regular Accelerated	11 (65%) 6 (35%)	20 (83%) 4 (17%)

Among the 13 RCTs with companion diagnostics (excluding studies in tumors with ubiquitous molecular alterations), 11 studies (85%) utilized prospective development of the diagnostic test in the RCT, while in 2 studies [11, 12] the companion diagnostic was developed retrospectively. In 11 RCTs (85%), the companion diagnostic comprised measurement of a mutation of which the protein product was an oncogenic target associated with favourable response to the experimental drug. Conversely, in 2 studies (15%) [11, 12] the diagnostic test identified patients with unfavourable responses to the investigational agent. Companion diagnostics associated with favourable drug responses comprised genomic alterations in non-small cell lung cancer (*EGFR* mutations and *ALK* rearrangements), breast and gastric cancers (*HER2/neu* amplification or overexpression), and in melanoma (*B-RAF* mutation). Companion diagnostics associated with resistance to the experimental drug were for mutations of the *K-RAS* gene in colon cancer.

### Impact of companion diagnostics on efficacy

**Figure 2:**
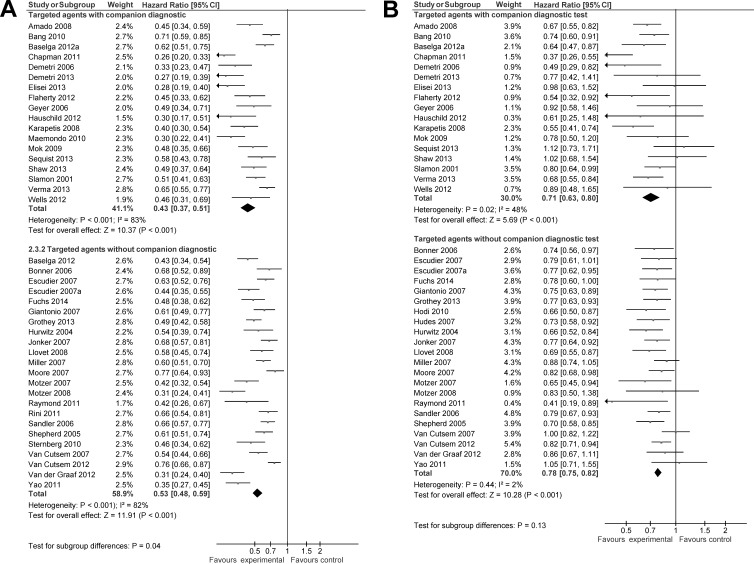
Forest plot for progression free survival (A) and overall survival (B)

Data on OS and PFS were available from 39 and 41 studies respectively. Compared to agents used in control groups, targeted drugs used in experimental arms of included trials showed improved PFS and OS. The magnitude of improvement in OS between targeted drugs in experimental groups and agents used in control groups was similar for targeted drugs with companion diagnostics and those without companion diagnostic tests (HR 0.71, 95% CI 0.63-0.80 *vs*. HR 0.78, 95% CI 0.75-0.82, p for difference = 0.13, Figure 2B). When studies for conditions with ubiquitous molecular alterations or mutations [7-10] were excluded, there remained no difference between studies with companion diagnostics and those without (HR 0.70, 95% CI 0.61-0.80 *vs*. HR 0.78, 95% CI 0.75-0.82, p for difference = 0.11).

In contrast, agents with an available companion diagnostic were associated with a higher magnitude of benefit for PFS compared to agents without companion diagnostic tests (HR 0.43, 95% CI 0.37-0.51 *vs*. 0.53, 95% CI 0.45-0.54, p for difference = 0.04, Figure 2A). However, this effect was lost after excluding agents used for conditions with ubiquitous activation of specific molecular pathways [7-10] (HR 0.47, 95% CI 0.40-0.55 *vs*. 0.53, 95% CI 0.45-0.54, p for difference = 0.20).

### Impact of companion diagnostics on safety and tolerability

**Figure 3:**
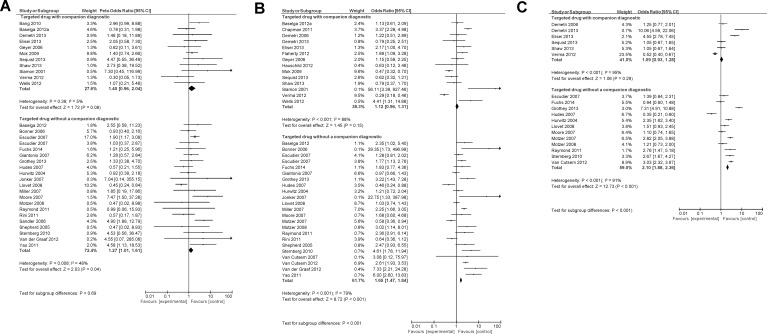
Forest plot for toxic death (A), treatment discontinuation (B) and grade 3/4 AEs (C)

Data on toxic death, treatment discontinuation and any grade 3/4 AEs were available from 32, 37 and 18 studies, respectively. Compared to agents used in control groups, targeted drugs used in experimental arms of included trials showed an increase in toxic death, treatment discontinuation and grade 3/4 AEs. Availability of companion diagnostics did not affect the magnitude of toxic death (OR 1.40, 95% CI 0.96-2.04 *vs*. 1.27, 95% CI 1.01-1.61, p for difference = 0.69, Figure 3A). However, availability of companion diagnostics was associated with lower odds of treatment discontinuation (OR 1.12, 95% CI 0.96-1.31 *vs*. 1.65, 95% CI 1.47-1.84, p for difference < 0.001, Figure 3B) and any grade 3/4 AEs (OR 1.09, 95% CI 0.93-1.28 *vs*. 2.10, 95% CI 1.88-2.36, p for difference < 0.001, Figure 3C). Differences between drugs with companion diagnostics and those without for individual grade 3/4 AEs are shown in Table 2. Companion diagnostics were associated with lower odds of anemia, neutropenia, diarrhea, vomiting, fatigue/asthenia, dyspnea, neuropathy and skin AEs; similar odds of stomatitis, hypertension, pulmonary and cardiovascular AEs and higher odds of thrombocytopenia. Sensitivity analyses restricting studies based on blinding and type of control group are shown in Table 3. Sensitivity analyses did not show substantial differences in the results. There remained no difference in the odds of toxic death between agents with companion diagnostics and those without in all subgroups. Furthermore, the magnitude and direction of effect for treatment discontinuation was similar among subgroups as it was in the analysis of all included studies. However, lower odds of grade 3/4 AEs were not observed among blinded studies or in placebo controlled only control groups. Of interest, among the minority of studies reporting quality of life, studies utilizing companion diagnostics were more likely to report improved quality of life compared to those with companion diagnostics (Mann-Whitney U *p* = 0.03).

**Table 2:** Differences in odds of individual grade 3/4 AEs between studies with available companion diagnostics and those without companion diagnostics AEs, adverse events, CI, confidence interval; OR, odds ratio

Adverse event	Companion Diagnostics			No Companion Diagnostics			P for difference
	Number of included studies	OR	95% CI	Number of included studies	OR	95% CI	
Anemia	8	0.79	0.62-1.00	12	1.14	0.89-1.46	0.04
Neutropenia	10	0.39	0.33-0.44	10	1.40	1.20-1.63	<0.001
Thrombocytopenia	5	5.18	3.09-8.66	12	1.66	1.23-2.22	<0.001
Diarrhea	14	1.29	1.07-1.55	22	2.43	2.08-2.83	<0.001
Vomiting	9	0.86	0.65-1.14	20	1.44	1.14-1.83	0.005
Stomatitis	7	1.53	0.78-3.02	14	2.59	2.01-3.33	0.16
Cutaneous toxicity	11	1.82	1.45-2.28	18	3.88	3.06-4.94	<0.001
Asthenia/Fatigue	14	0.99	0.79-1.23	24	1.23	1.11-1.37	0.07
Pulmonary/Dyspnea	6	1.15	0.84-1.57	11	0.90	0.74-1.09	0.19
Hypertension	5	5.95	3.03-11.67	14	5.87	4.74-7.26	0.97
Cardiac	4	1.57	1.11-2.23	3	6.88	1.25-37.82	0.10
Neuropathy	3	0.64	0.40-1.01	3	1.60	1.20-2.12	<0.001

**Table 3:** Sensitivity analyses for the effect of availability of companion diagnostics on adverse events AEs, adverse events; CI, confidence interval; N, number; OR, odds ratio. NE, not estimatable

Group	N	Companion Diagnostic		Non-Companion Diagnostic		P for difference
		OR	95% CI	OR	95% CI	
Toxic death						
Blinded companion [8-10, 24] vs non-Companion [25-38]	18	1.12	0.59 - 2.14	1.29	0.97 - 1.72	0.69
Open-label companion [39-45] vs non-Companion [46-52]	14	1.57	0.98 - 2.51	1.24	0.82 - 1.86	0.46
Best supportive care companion vs non-Companion [46, 47]	2	NE	NE	1.02	0.45 - 2.31	NE
Placebo-control only control group companion [8-10] vs non-Companion [26-28, 30, 32, 33] [34-37]	13	1.58	0.64 - 3.9	1.25	0.89 - 1.74	0.63
Active Treatment control group companion [24, 39-45] vs non-Companion [25-26, 29, 31, 48-52]	17	1.36	0.9 - 2.07	1.36	0.95 - 1.96	0.99
Active Treatment + Placebo control group companion [24] vs non-Companion [25-26, 29, 31]	4	0.78	0.31 - 1.98	1.3	0.7 - 2.39	0.37
Treatment discontinuation						
Blinded companion [7-10, 24] vs non-Companion [25-38, 53]	20	1.57	1.09 - 2.26	1.97	1.71 - 2.28	0.25
Open-label companion[40-45, 54-56] vs non-Companion[46-51, 57-58]	17	1.04	0.87 - 1.23	1.22	1.02 - 1.47	0.19
Best supportive care companion vs non-Companion [46-47, 58]	3	NE	NE	18.18	3.51 - 94.19	NE
Placebo-control only control group companion [8-10], vs non-Companion [26-28, 30, 32-37]	14	1.85	1.16 - 2.94	1.90	1.56 - 2.32	0.91
Active Treatment control group companion [24, 40-45, 54-56] vs non-Companion [25, 29, 31, 38, 48-51, 53, 57]	20	1.04	0.88 - 1.23	1.46	1.27 - 1.68	0.002
Active Treatment + Placebo control group companion [24] vs non-Companion [25-26, 29, 31]	6	1.13	0.61 - 2.09	2.06	1.67 - 2.54	0.07
Any G3/4 AEs						
Blinded companion [7-9] vs non-Companion [26-33, 35, 53]	13	3.05	2.26 - 4.12	2.26	1.99 - 2.57	0.07
Open-label companion [42-43, 45] vs non-Companion [49, 57]	5	0.68	0.56 - 0.83	1.57	1.21 - 2.03	<0.001
Best supportive care companion vs non-Companion	0	NE	NE	NE	NE	NE
Placebo-control only control group companion [7-9] vs non-Companion [26-28, 30, 32-33, 35]	10	3.05	2.26 - 4.12	2.26	1.9 - 2.69	0.09
Active Treatment control group companion [42-43, 45] vs non-Companion [29, 31, 49, 53, 57]	8	0.68	0.56 - 0.83	1.99	1.71 - 2.32	<0.001
Active Treatment + Placebo control group companion vs non-Companion [29, 31]	3	NE	NE	2.26	1.87 - 2.73	NE

## DISCUSSION

The presence of heterogeneity within histologically defined tumors means that the ability to identify subpopulations of patients who are more likely to benefit from a certain treatment is crucial [13]. Advances in the understanding of cancer biology have permitted the identification of molecular alterations that can be used for patient selection; both by ascertaining molecular alterations that are amenable to pharmacological intervention or by characterizing markers of resistance to specific therapy. In recent years, numerous targeted drugs have been approved for use in solid tumors. Some of these were approved with an accompanying companion diagnostic test allowing for identification of responsive or unresponsive patients while others were registered in less selected populations. Despite the increasing use of targeted agents and diagnostic tests to aid in personalization of therapy, there remain little data to inform of the impact of the availability of companion diagnostics on the efficacy, safety and tolerability of approved targeted anti-cancer drugs. Specifically, it is unclear if targeted agents with available companion diagnostics allow for both improved efficacy and toxicity compared to targeted agents used in unselected cancer patients.

As expected, we have shown that availability of companion diagnostics is associated with modest improvements in efficacy of targeted anti-cancer drugs. Compared to treatment administered in control groups of RCTs, use of experimental targeted treatment with a companion diagnostic led to greater improvements in PFS than for experimental targeted agents without accompanying tests. However, the availability of companion diagnostics did not appear to influence the magnitude of benefit in OS; targeted agents with and without these tests showed similar improvements in pooled HR for OS.

Of interest, our data showed generally better safety and tolerability for experimental targeted treatment with a companion diagnostic compared to targeted agents without accompanying tests. Consistent with data reported previously [14, 15], we observed that newly approved targeted drugs were generally associated with higher odds of toxic death, treatment discontinuation due to toxicity and both overall grade 3/4 AEs as well as individual grade 3/4 AEs. However, availability of companion diagnostics was associated with a lower magnitude of increase in the majority of these safety and tolerability parameters. Additionally, studies with companion diagnostics were more likely to report favorable changes in quality of life. These results suggest that targeted drugs developed with companion diagnostics may have a more favorable therapeutic index than targeted drugs without such accompanying tests. There are a number of possible reasons for this finding. First, the availability of a companion diagnostic may allow for lower doses of targeted drugs to be used in more responsive patients. Second, receipt of more efficacious drugs may lead to reduced tumor burden or improved performance status thereby leading to a lower frequency of AEs. Of note, differences in grade 3/4 AEs between targeted drugs with companion diagnostics and targeted drugs without these tests were not consistently observed in both blinded and open-label studies suggesting that some of the effect may relate to either subject or observer bias. However, no such findings were observed for treatment discontinuation suggesting that some of the differences in safety and tolerability parameters are likely true.

The availability of a companion diagnostic was also associated with differences in clinical trial design. RCTs utilizing companion diagnostics had smaller sample sizes. The reason for this finding is unclear, but may be explained by the greater magnitude of anticipated benefit resulting from enriching the study population with patients more likely to benefit from the experimental drug. Another explanation is the increased use of intermediate endpoints such as PFS, which was seen with studies utilizing companion diagnostics. No differences were observed for other parameters such as whether RCTs were used to support accelerated rather than regular approval, allowance of cross over or reporting of quality of life.

The value of companion diagnostics as predictive markers appears clear. However, only a minority of approved targeted drugs have been developed with such diagnostic tests. The development of companion diagnostics in solid tumors is complex and has many challenges. Access to tissue for molecular characterization is guaranteed generally only at diagnosis and attempts to collect tissue at multiple time points after exposure to experimental therapy are limited by concerns about slower patient enrolment and insufficient research infrastructure to obtain and process tumor samples for molecular analysis. There has also been reluctance of the scientific community to design studies with mandatory repeated tissue collection [16]. This may explain why many biomarker programs embedded in RCTs do not lead to the identification of robust predictive markers.

This study has limitations. First we focused on RCTs, which were used to support registration with the US FDA. While this is a pragmatic and clinically relevant inclusion criterion, there may have been negative studies with companion diagnostics and their exclusion may have affected the observed difference in effect size for efficacy, safety and tolerability. It is likely that this may have led to enrichment for agents with a more favorable balance between efficacy and toxicity. Second, assessment of efficacy was based only on relative differences in OS or PFS between groups. Substantial differences in relative measures of efficacy do not necessarily translate into large differences in absolute benefits. Third, only 18 studies provided information on number of patients experiencing at least one grade 3/4 AE, although assessment of individual toxicities yielded similar results especially for non-hematological AEs. Finally, all included studies were designed to test primarily the efficacy of new systemic treatments and the outcome measures included in our analysis of toxicity were not primary endpoints in any of the included studies. Collection and reporting of toxicity data in published RCTs can be suboptimal and variable [17-19] so that results of our analysis may not represent the true rates of toxicity even within the included studies.

In conclusion, in addition to improvements in PFS, targeted drugs with companion diagnostics are associated with improved safety, and tolerability.

## MATERIALS AND METHODS

This analysis was conducted in accordance with the Preferred Reporting Items for Systematic Reviews and Meta-Analyses (PRISMA) guidelines. PRISMA checklist is shown in Supplementary Figure 1.

### Identification of studies

All targeted, anticancer agents approved by the US Food and Drug administration (FDA) between January 2000 and April 2014 for treatment of advanced or metastatic solid malignancies in adults were identified through the FDA website [20]. We excluded agents used in supportive cancer care, such as antiemetic drugs or bisphosphonates. We also excluded studies of cytotoxic chemotherapy, systemic radiation therapy, endocrine therapy and cancer vaccines. Drug labels of included agents were consulted for all referenced RCTs. We reviewed each article and identified those that used a companion diagnostic to select patients. We also reviewed the FDA website to evaluate those companion diagnostic tests approved for the specific indication.

### Data extraction

Extracted data included the experimental agent, the diagnostic kit used in the study, the molecular alteration evaluated, tumor type, primary endpoint, use of cross-over, type of FDA approval (regular or accelerated), reporting of quality of life, and year of publication. For efficacy outcomes, we extracted hazard ratios (HR) and their corresponding 95% confidence intervals (CI) for time-to-event endpoints including overall survival (OS) and/or progression-free survival (PFS). When data for PFS were not available, we extracted data for time to progression (TTP). For toxicity outcomes, the events of interest were the occurrence of death reported as being related to systemic treatment, treatment-discontinuation related to AEs and occurrence of grade 3/4 AEs. If a drug label did not report toxic death, it was assumed to be zero as such events are mandated to be reported to the FDA. For AEs, the number or proportion of patients with at least one grade 3/4 AE was collected. The 12 most commonly reported AEs were also assessed individually and included anemia, neutropenia, thrombocytopenia, diarrhea, vomiting, stomatitis, hypertension, fatigue/asthenia, neuropathy as well as cardiovascular, pulmonary and skin AEs. Data were extracted by three authors (JE, VS and LD). Discrepancies were resolved by consensus.

### Data synthesis

Drugs were assigned to one of two subgroups: targeted agents without companion diagnostic and targeted agents with available companion diagnostic. For the primary analysis, tumors with ubiquitous molecular alterations or mutations (e.g. *c-kit* in GIST or *RET* in medullary thyroid cancer) were included in the group of studies with companion diagnostics. To identify whether different levels of efficacy were derived from availability of companion diagnostics, we pooled HRs for OS and PFS for all available studies. A sensitivity analysis was conducted excluding studies conducted in conditions with ubiquitous activation of specific molecular pathways. For toxicity, we compared the rates of treatment-related death, treatment discontinuation due to toxicity and grade 3/4 AEs between the companion diagnostic and non-companion diagnostic groups. Sensitivity analyses were conducted to examine the effect of blinding (i.e. blinded or open-label studies) and type of control group (placebo-only, active treatment or active treatment plus placebo) on toxicity outcomes.

### Statistical analysis

Data were reported descriptively as means or medians together with respective standard deviations and ranges, respectively. When possible, data were combined into a meta-analysis using RevMan 5.3 analysis software (Cochrane Collaboration, Copenhagen, Denmark). Pooled estimates of HRs for OS and PFS were weighed by generic inverse variance and computed by random effects modeling [21, 22]. Pooled estimates of odds ratios (OR) of safety and tolerability endpoints were computed using different methods for toxic death, treatment-discontinuation and grade 3/4 AEs. For toxic death where absolute event rates were less than 1%, the Peto one-step odds ratio method was utilized [23]. For treatment-discontinuation where there were low absolute event rates and substantial variability in relative effect-sizes, the Mantel-Haenszel odds ratio method was used [21]. Finally, for grade 3/4 AEs, the DerSimonian and Laird random-effects method was utilized and studies were weighted using the generic inverse variance approach [21, 22]. Differences in the pooled estimates for companion and on-companion studies were evaluated using subgroup analysis as described by Deeks et al [21]. All statistical tests were two-sided, and statistical significance was defined as *p* < 0.05. No corrections were made for multiple testing.

## SUPPLEMENTARY MATERIAL TABLES



## References

[1] Wood LD, Parsons DW, Jones S, Lin J, Sjoblom T, Leary RJ, Shen D, Boca SM, Barber T, Ptak J, Silliman N, Szabo S, Dezso Z, Ustyanksky V, Nikolskaya T, Nikolsky Y (2007). The genomic landscapes of human breast and colorectal cancers. Science.

[2] Vogelstein B, Papadopoulos N, Velculescu VE, Zhou S, Diaz LA (2013). and Kinzler KW. Cancer genome landscapes. Science.

[3] Kris MG, Johnson BE, Berry LD, Kwiatkowski DJ, Iafrate AJ, Wistuba II, Varella-Garcia M, Franklin WA, Aronson SL, Su PF, Shyr Y, Camidge DR, Sequist LV, Glisson BS, Khuri FR, Garon EB (2014). Using multiplexed assays of oncogenic drivers in lung cancers to select targeted drugs. Jama.

[4] Ocana A, Pandiella A (2010). Personalized therapies in the cancer “omics” era. Molecular cancer.

[5] Rubin EH, Allen JD, Nowak JA, Bates SE (2014). Developing precision medicine in a global world. Clinical cancer research : an official journal of the American Association for Cancer Research.

[6] Mansfield EA (2014). FDA perspective on companion diagnostics: an evolving paradigm. Clinical cancer research : an official journal of the American Association for Cancer Research.

[7] Demetri GD, van Oosterom AT, Garrett CR, Blackstein ME, Shah MH, Verweij J, McArthur G, Judson IR, Heinrich MC, Morgan JA, Desai J, Fletcher CD, George S, Bello CL, Huang X, Baum CM (2006). Efficacy and safety of sunitinib in patients with advanced gastrointestinal stromal tumour after failure of imatinib: a randomised controlled trial. Lancet.

[8] Demetri GD, Reichardt P, Kang YK, Blay JY, Rutkowski P, Gelderblom H, Hohenberger P, Leahy M, von Mehren M, Joensuu H, Badalamenti G, Blackstein M, Le Cesne A, Schoffski P, Maki RG, Bauer S (2013). Efficacy and safety of regorafenib for advanced gastrointestinal stromal tumours after failure of imatinib and sunitinib (GRID): an international, multicentre, randomised, placebo-controlled, phase 3 trial. Lancet.

[9] Elisei R, Schlumberger MJ, Muller SP, Schoffski P, Brose MS, Shah MH, Licitra L, Jarzab B, Medvedev V, Kreissl MC, Niederle B, Cohen EE, Wirth LJ, Ali H, Hessel C, Yaron Y (2013). Cabozantinib in progressive medullary thyroid cancer. Journal of clinical oncology : official journal of the American Society of Clinical Oncology.

[10] Wells SA, Robinson BG, Gagel RF, Dralle H, Fagin JA, Santoro M, Baudin E, Elisei R, Jarzab B, Vasselli JR, Read J, Langmuir P, Ryan AJ, Schlumberger MJ (2012). Vandetanib in patients with locally advanced or metastatic medullary thyroid cancer: a randomized, double-blind phase III trial. Journal of clinical oncology : official journal of the American Society of Clinical Oncology.

[11] Karapetis CS, Khambata-Ford S, Jonker DJ, O'Callaghan CJ, Tu D, Tebbutt NC, Simes RJ, Chalchal H, Shapiro JD, Robitaille S, Price TJ, Shepherd L, Au HJ, Langer C, Moore MJ, Zalcberg JR (2008). K-ras mutations and benefit from cetuximab in advanced colorectal cancer. The New England journal of medicine.

[12] Amado RG, Wolf M, Peeters M, Van Cutsem E, Siena S, Freeman DJ, Juan T, Sikorski R, Suggs S, Radinsky R, Patterson SD, Chang DD (2008). Wild-type KRAS is required for panitumumab efficacy in patients with metastatic colorectal cancer. Journal of clinical oncology : official journal of the American Society of Clinical Oncology.

[13] Gerlinger M, Rowan AJ, Horswell S, Larkin J, Endesfelder D, Gronroos E, Martinez P, Matthews N, Stewart A, Tarpey P, Varela I, Phillimore B, Begum S, McDonald NQ, Butler A, Jones D (2012). Intratumor heterogeneity and branched evolution revealed by multiregion sequencing. The New England journal of medicine.

[14] Niraula S, Amir E, Vera-Badillo F, Seruga B, Ocana A, Tannock IF (2014). Risk of incremental toxicities and associated costs of new anticancer drugs: a meta-analysis. Journal of clinical oncology : official journal of the American Society of Clinical Oncology.

[15] Niraula S, Seruga B, Ocana A, Shao T, Goldstein R, Tannock IF, Amir E (2012). The price we pay for progress: a meta-analysis of harms of newly approved anticancer drugs. Journal of clinical oncology : official journal of the American Society of Clinical Oncology.

[16] Sawyers CL (2008). The cancer biomarker problem. Nature.

[17] Seruga B, Sterling L, Wang L, Tannock IF (2011). Reporting of serious adverse drug reactions of targeted anticancer agents in pivotal phase III clinical trials. Journal of clinical oncology : official journal of the American Society of Clinical Oncology.

[18] Pitrou I, Boutron I, Ahmad N, Ravaud P (2009). Reporting of safety results in published reports of randomized controlled trials. Archives of internal medicine.

[19] Ioannidis JP (2009). Adverse events in randomized trials: neglected, restricted, distorted, and silenced. Archives of internal medicine.

[20] http://www.accessdata.fda.gov/Scripts/cder/drugsatfda/index.cfm.

[21] Deeks J.J., Higgins J.P.T., Altman D.G. (2006). Analysing and presenting results. Cochrane Handbook for Systematic Reviews of Interventions 4 2 5.

[22] DerSimonian R, Laird N (1986). Meta-analysis in clinical trials. Controlled clinical trials.

[23] Sweeting MJ, Sutton AJ, Lambert PC (2004). What to add to nothing? Use and avoidance of continuity corrections in meta-analysis of sparse data. Statistics in medicine.

[24] Baselga J, Cortes J, Kim SB, Im SA, Hegg R, Im YH, Roman L, Pedrini JL, Pienkowski T, Knott A, Clark E, Benyunes MC, Ross G, Swain SM, Group CS (2012). Pertuzumab plus trastuzumab plus docetaxel for metastatic breast cancer. The New England journal of medicine.

[25] Baselga J, Campone M, Piccart M, Burris HA, Rugo HS, Sahmoud T, Noguchi S, Gnant M, Pritchard KI, Lebrun F, Beck JT, Ito Y, Yardley D, Deleu I, Perez A, Bachelot T (2012). Everolimus in postmenopausal hormone-receptor-positive advanced breast cancer. The New England journal of medicine.

[26] Escudier B, Eisen T, Stadler WM, Szczylik C, Oudard S, Siebels M, Negrier S, Chevreau C, Solska E, Desai AA, Rolland F, Demkow T, Hutson TE, Gore M, Freeman S, Schwartz B (2007). Sorafenib in advanced clear-cell renal-cell carcinoma. The New England journal of medicine.

[27] Fuchs CS, Tomasek J, Yong CJ, Dumitru F, Passalacqua R, Goswami C, Safran H, dos Santos LV, Aprile G, Ferry DR, Melichar B, Tehfe M, Topuzov E, Zalcberg JR, Chau I, Campbell W (2014). Ramucirumab monotherapy for previously treated advanced gastric or gastro-oesophageal junction adenocarcinoma (REGARD): an international, randomised, multicentre, placebo-controlled, phase 3 trial. Lancet.

[28] Grothey A, Van Cutsem E, Sobrero A, Siena S, Falcone A, Ychou M, Humblet Y, Bouche O, Mineur L, Barone C, Adenis A, Tabernero J, Yoshino T, Lenz HJ, Goldberg RM, Sargent DJ (2013). Regorafenib monotherapy for previously treated metastatic colorectal cancer (CORRECT): an international, multicentre, randomised, placebo-controlled, phase 3 trial. Lancet.

[29] Hurwitz H, Fehrenbacher L, Novotny W, Cartwright T, Hainsworth J, Heim W, Berlin J, Baron A, Griffing S, Holmgren E, Ferrara N, Fyfe G, Rogers B, Ross R, Kabbinavar F (2004). Bevacizumab plus irinotecan, fluorouracil, and leucovorin for metastatic colorectal cancer. The New England journal of medicine.

[30] Llovet JM, Ricci S, Mazzaferro V, Hilgard P, Gane E, Blanc JF, de Oliveira AC, Santoro A, Raoul JL, Forner A, Schwartz M, Porta C, Zeuzem S, Bolondi L, Greten TF, Galle PR (2008). Sorafenib in advanced hepatocellular carcinoma. The New England journal of medicine.

[31] Moore MJ, Goldstein D, Hamm J, Figer A, Hecht JR, Gallinger S, Au HJ, Murawa P, Walde D, Wolff RA, Campos D, Lim R, Ding K, Clark G, Voskoglou-Nomikos T, Ptasynski M (2007). Erlotinib plus gemcitabine compared with gemcitabine alone in patients with advanced pancreatic cancer: a phase III trial of the National Cancer Institute of Canada Clinical Trials Group. Journal of clinical oncology : official journal of the American Society of Clinical Oncology.

[32] Motzer RJ, Escudier B, Oudard S, Hutson TE, Porta C, Bracarda S, Grunwald V, Thompson JA, Figlin RA, Hollaender N, Urbanowitz G, Berg WJ, Kay A, Lebwohl D, Ravaud A, Group R-S (2008). Efficacy of everolimus in advanced renal cell carcinoma: a double-blind, randomised, placebo-controlled phase III trial. Lancet.

[33] Raymond E, Dahan L, Raoul JL, Bang YJ, Borbath I, Lombard-Bohas C, Valle J, Metrakos P, Smith D, Vinik A, Chen JS, Horsch D, Hammel P, Wiedenmann B, Van Cutsem E, Patyna S (2011). Sunitinib malate for the treatment of pancreatic neuroendocrine tumors. The New England journal of medicine.

[34] Shepherd FA, Rodrigues Pereira J, Ciuleanu T, Tan EH, Hirsh V, Thongprasert S, Campos D, Maoleekoonpiroj S, Smylie M, Martins R, van Kooten M, Dediu M, Findlay B, Tu D, Johnston D, Bezjak A (2005). Erlotinib in previously treated non-small-cell lung cancer. The New England journal of medicine.

[35] Sternberg CN, Davis ID, Mardiak J, Szczylik C, Lee E, Wagstaff J, Barrios CH, Salman P, Gladkov OA, Kavina A, Zarba JJ, Chen M, McCann L, Pandite L, Roychowdhury DF, Hawkins RE (2010). Pazopanib in locally advanced or metastatic renal cell carcinoma: results of a randomized phase III trial. Journal of clinical oncology : official journal of the American Society of Clinical Oncology.

[36] van der Graaf WT, Blay JY, Chawla SP, Kim DW, Bui-Nguyen B, Casali PG, Schoffski P, Aglietta M, Staddon AP, Beppu Y, Le Cesne A, Gelderblom H, Judson IR, Araki N, Ouali M, Marreaud S (2012). Pazopanib for metastatic soft-tissue sarcoma (PALETTE): a randomised, double-blind, placebo-controlled phase 3 trial. Lancet.

[37] Yao JC, Shah MH, Ito T, Bohas CL, Wolin EM, Van Cutsem E, Hobday TJ, Okusaka T, Capdevila J, de Vries EG, Tomassetti P, Pavel ME, Hoosen S, Haas T, Lincy J, Lebwohl D (2011). Everolimus for advanced pancreatic neuroendocrine tumors. The New England journal of medicine.

[38] Escudier B, Pluzanska A, Koralewski P, Ravaud A, Bracarda S, Szczylik C, Chevreau C, Filipek M, Melichar B, Bajetta E, Gorbunova V, Bay JO, Bodrogi I, Jagiello-Gruszfeld A, Moore N, investigators AT (2007). Bevacizumab plus interferon alfa-2a for treatment of metastatic renal cell carcinoma: a randomised, double-blind phase III trial. Lancet.

[39] Bang YJ, Van Cutsem E, Feyereislova A, Chung HC, Shen L, Sawaki A, Lordick F, Ohtsu A, Omuro Y, Satoh T, Aprile G, Kulikov E, Hill J, Lehle M, Ruschoff J, Kang YK (2010). Trastuzumab in combination with chemotherapy versus chemotherapy alone for treatment of HER2-positive advanced gastric or gastro-oesophageal junction cancer (ToGA): a phase 3, open-label, randomised controlled trial. Lancet.

[40] Geyer CE, Forster J, Lindquist D, Chan S, Romieu CG, Pienkowski T, Jagiello-Gruszfeld A, Crown J, Chan A, Kaufman B, Skarlos D, Campone M, Davidson N, Berger M, Oliva C, Rubin SD (2006). Lapatinib plus capecitabine for HER2-positive advanced breast cancer. The New England journal of medicine.

[41] Mok TS, Wu YL, Thongprasert S, Yang CH, Chu DT, Saijo N, Sunpaweravong P, Han B, Margono B, Ichinose Y, Nishiwaki Y, Ohe Y, Yang JJ, Chewaskulyong B, Jiang H, Duffield EL (2009). Gefitinib or carboplatin-paclitaxel in pulmonary adenocarcinoma. The New England journal of medicine.

[42] Sequist LV, Yang JC, Yamamoto N, O'Byrne K, Hirsh V, Mok T, Geater SL, Orlov S, Tsai CM, Boyer M, Su WC, Bennouna J, Kato T, Gorbunova V, Lee KH, Shah R (2013). Phase III study of afatinib or cisplatin plus pemetrexed in patients with metastatic lung adenocarcinoma with EGFR mutations. Journal of clinical oncology : official journal of the American Society of Clinical Oncology.

[43] Shaw AT, Kim DW, Nakagawa K, Seto T, Crino L, Ahn MJ, De Pas T, Besse B, Solomon BJ, Blackhall F, Wu YL, Thomas M, O'Byrne KJ, Moro-Sibilot D, Camidge DR, Mok T (2013). Crizotinib versus chemotherapy in advanced ALK-positive lung cancer. The New England journal of medicine.

[44] Slamon DJ, Leyland-Jones B, Shak S, Fuchs H, Paton V, Bajamonde A, Fleming T, Eiermann W, Wolter J, Pegram M, Baselga J, Norton L (2001). Use of chemotherapy plus a monoclonal antibody against HER2 for metastatic breast cancer that overexpresses HER2. The New England journal of medicine.

[45] Verma S, Miles D, Gianni L, Krop IE, Welslau M, Baselga J, Pegram M, Oh DY, Dieras V, Guardino E, Fang L, Lu MW, Olsen S, Blackwell K, Group ES (2012). Trastuzumab emtansine for HER2-positive advanced breast cancer. The New England journal of medicine.

[46] Bonner JA, Harari PM, Giralt J, Azarnia N, Shin DM, Cohen RB, Jones CU, Sur R, Raben D, Jassem J, Ove R, Kies MS, Baselga J, Youssoufian H, Amellal N, Rowinsky EK (2006). Radiotherapy plus cetuximab for squamous-cell carcinoma of the head and neck. The New England journal of medicine.

[47] Jonker DJ, O'Callaghan CJ, Karapetis CS, Zalcberg JR, Tu D, Au HJ, Berry SR, Krahn M, Price T, Simes RJ, Tebbutt NC, van Hazel G, Wierzbicki R, Langer C, Moore MJ (2007). Cetuximab for the treatment of colorectal cancer. The New England journal of medicine.

[48] Giantonio BJ, Catalano PJ, Meropol NJ, O'Dwyer PJ, Mitchell EP, Alberts SR, Schwartz MA, Benson AB, Eastern Cooperative Oncology Group Study E (2007). Bevacizumab in combination with oxaliplatin, fluorouracil, and leucovorin (FOLFOX4) for previously treated metastatic colorectal cancer: results from the Eastern Cooperative Oncology Group Study E3200. Journal of clinical oncology : official journal of the American Society of Clinical Oncology.

[49] Hudes G, Carducci M, Tomczak P, Dutcher J, Figlin R, Kapoor A, Staroslawska E, Sosman J, McDermott D, Bodrogi I, Kovacevic Z, Lesovoy V, Schmidt-Wolf IG, Barbarash O, Gokmen E, O'Toole T (2007). Temsirolimus, interferon alfa, or both for advanced renal-cell carcinoma. The New England journal of medicine.

[50] Miller AA, Wang XF, Bogart JA, Hodgson LD, Rocha Lima CM, Radford JE, Vokes EE, Green MR, Cancer Leukemia Group B (2007). Phase II trial of paclitaxel-topotecan-etoposide followed by consolidation chemoradiotherapy for limited-stage small cell lung cancer: CALGB 30002. Journal of thoracic oncology : official publication of the International Association for the Study of Lung Cancer.

[51] Rini BI, Escudier B, Tomczak P, Kaprin A, Szczylik C, Hutson TE, Michaelson MD, Gorbunova VA, Gore ME, Rusakov IG, Negrier S, Ou YC, Castellano D, Lim HY, Uemura H, Tarazi J (2011). Comparative effectiveness of axitinib versus sorafenib in advanced renal cell carcinoma (AXIS): a randomised phase 3 trial. Lancet.

[52] Sandler A, Gray R, Perry MC, Brahmer J, Schiller JH, Dowlati A, Lilenbaum R, Johnson DH (2006). Paclitaxel-carboplatin alone or with bevacizumab for non-small-cell lung cancer. The New England journal of medicine.

[53] Van Cutsem E, Tabernero J, Lakomy R, Prenen H, Prausova J, Macarulla T, Ruff P, van Hazel GA, Moiseyenko V, Ferry D, McKendrick J, Polikoff J, Tellier A, Castan R, Allegra C (2012). Addition of aflibercept to fluorouracil, leucovorin, and irinotecan improves survival in a phase III randomized trial in patients with metastatic colorectal cancer previously treated with an oxaliplatin-based regimen. Journal of clinical oncology : official journal of the American Society of Clinical Oncology.

[54] Chapman PB, Hauschild A, Robert C, Haanen JB, Ascierto P, Larkin J, Dummer R, Garbe C, Testori A, Maio M, Hogg D, Lorigan P, Lebbe C, Jouary T, Schadendorf D, Ribas A (2011). Improved survival with vemurafenib in melanoma with BRAF V600E mutation. The New England journal of medicine.

[55] Flaherty KT, Puzanov I, Kim KB, Ribas A, McArthur GA, Sosman JA, O'Dwyer PJ, Lee RJ, Grippo JF, Nolop K, Chapman PB (2010). Inhibition of mutated, activated BRAF in metastatic melanoma. The New England journal of medicine.

[56] Hauschild A, Grob JJ, Demidov LV, Jouary T, Gutzmer R, Millward M, Rutkowski P, Blank CU, Miller WH, Kaempgen E, Martin-Algarra S, Karaszewska B, Mauch C, Chiarion-Sileni V, Martin AM, Swann S (2012). Dabrafenib in BRAF-mutated metastatic melanoma: a multicentre, open-label, phase 3 randomised controlled trial. Lancet.

[57] Motzer RJ, Hutson TE, Tomczak P, Michaelson MD, Bukowski RM, Rixe O, Oudard S, Negrier S, Szczylik C, Kim ST, Chen I, Bycott PW, Baum CM, Figlin RA (2007). Sunitinib versus interferon alfa in metastatic renal-cell carcinoma. The New England journal of medicine.

[58] Van Cutsem E, Peeters M, Siena S, Humblet Y, Hendlisz A, Neyns B, Canon JL, Van Laethem JL, Maurel J, Richardson G, Wolf M, Amado RG (2007). Open-label phase III trial of panitumumab plus best supportive care compared with best supportive care alone in patients with chemotherapy-refractory metastatic colorectal cancer. Journal of clinical oncology: official journal of the American Society of Clinical Oncology.

