# Supplementary material for: Influence of companion diagnostics on efficacy and safety of targeted anti-cancer drugs: systematic review and meta-analyses
Source: Oncotarget. 2015 Oct 4;6(37):39538–49. doi: 10.18632/oncotarget.5946 (PMC4741844; doi:10.18632/oncotarget.5946)
Supplement: Supplementary file 1 [file oncotarget-06-39538-s001.pdf]

Influence of companion diagnostics on efficacy and safety of targeted anti-cancer drugs: systematic review and meta-analyses

Supplementary Material

Section/topic	#	Checklist item	Reported on page #
**TITLE**			
Title	1	Identify the report as a systematic review, meta-analysis, or both.	1
**ABSTRACT**			
Structured summary	2	Provide a structured summary including, as applicable: background; objectives; data sources; study eligibility criteria, participants, and interventions; study appraisal and synthesis methods; results; limitations; conclusions and implications of key findings; systematic review registration number.	1
**INTRODUCTION**			2
Rationale	3	Describe the rationale for the review in the context of what is already known.	
Objectives	4	Provide an explicit statement of questions being addressed with reference to participants, interventions, comparisons, outcomes, and study design (PICOS).	
**METHODS**			7-8
Protocol and registration	5	Indicate if a review protocol exists, if and where it can be accessed (e.g., Web address), and, if available, provide registration information including registration number.	
Eligibility criteria	6	Specify study characteristics (e.g., PICOS, length of follow-up) and report characteristics (e.g., years considered, language, publication status) used as criteria for eligibility, giving rationale.	
Information sources	7	Describe all information sources (e.g., databases with dates of coverage, contact with study authors to identify additional studies) in the search and date last searched.	
Search	8	Present full electronic search strategy for at least one database, including any limits used, such that it could be repeated.	
Study selection	9	State the process for selecting studies (i.e., screening, eligibility, included in systematic review, and, if applicable, included in the meta-analysis).	
Data collection process	10	Describe method of data extraction from reports (e.g., piloted forms, independently, in duplicate) and any processes for obtaining and confirming data from investigators.	
Data items	11	List and define all variables for which data were sought (e.g., PICOS, funding sources) and any assumptions and simplifications made.	
Risk of bias in individual studies	12	Describe methods used for assessing risk of bias of individual studies (including specification of whether this was done at the study or outcome level), and how this information is to be used in any data synthesis.	
Summary measures	13	State the principal summary measures (e.g., risk ratio, difference in means).	
Synthesis of results	14	Describe the methods of handling data and combining results of studies, if done, including measures of consistency (e.g., I^2) for each meta-analysis.	
Risk of bias across studies	15	Specify any assessment of risk of bias that may affect the cumulative evidence (e.g., publication bias, selective reporting within studies).	
Additional analyses	16	Describe methods of additional analyses (e.g., sensitivity or subgroup analyses, meta-regression), if done, indicating which were pre-specified.	
**RESULTS**			2
Study selection	17	Give numbers of studies screened, assessed for eligibility, and included in the review, with reasons for exclusions at each stage, ideally with a flow diagram.	
Study characteristics	18	For each study, present characteristics for which data were extracted (e.g., study size, PICOS, follow-up period) and provide the citations.	
Risk of bias within studies	19	Present data on risk of bias of each study and, if available, any outcome level assessment (see item 12).	
Results of individual studies	20	For all outcomes considered (benefits or harms), present, for each study: (a) simple summary data for each intervention group (b) effect estimates and confidence intervals, ideally with a forest plot.	
Synthesis of results	21	Present results of each meta-analysis done, including confidence intervals and measures of consistency.	
Risk of bias across studies	22	Present results of any assessment of risk of bias across studies (see Item 15).	
Additional analysis	23	Give results of additional analyses, if done (e.g., sensitivity or subgroup analyses, meta-regression [see Item 16]).	
**DISCUSSION**			6-7
Summary of evidence	24	Summarize the main findings including the strength of evidence for each main outcome; consider their relevance to key groups (e.g., healthcare providers, users, and policy makers).	
Limitations	25	Discuss limitations at study and outcome level (e.g., risk of bias), and at review-level (e.g., incomplete retrieval of identified research, reporting bias).	
Conclusions	26	Provide a general interpretation of the results in the context of other evidence, and implications for future research.	
**FUNDING**			8
Funding	27	Describe sources of funding for the systematic review and other support (e.g., supply of data); role of funders for the systematic review.	

Supplementary Table 1. FDA approved drugs by indication in adult solid tumor.

Tumor Type	Study Drugs
Breast Cancer	**Targeted agents non companion diagnostics**<br>•Bevacizumab, Everolimus<br>**Targeted agents companion diagnostics**<br>•Lapatinib, Pertuzumab, Trastuzumab, Trastuzumab emtansine
Colorectal Cancer	**Targeted agents non companion diagnostics**<br>•Aflibercept<br>•Bevacizumab<br>•Cetuximab<br>•Panitumumab<br>•Regorafenib<br>**Targeted agents companion diagnostics**<br>•Cetuximab<br>•Panitumumab
Gastric or Gastro-oesophageal Cancer	**Targeted agents non companion diagnostics**<br>•Ramucirumab<br>**Targeted agents companion diagnostics**<br>•Trastuzumab

Gastrointestinal Stromal Tumor	**Target agents against a tumor with a ubiquitous molecular alteration**<br>•Regorafenib<br>•Sunitinib
Hepatocellular Carcinoma	**Targeted agents non companion diagnostics**<br>•Sorafenib
Head and Neck Cancer	**Targeted agents non companion diagnostics**<br>•Cetuximab
Lung Cancer	**Targeted agents non companion diagnostics**<br>•Bevacizumab<br>•Erlotinib<br>**Targeted agents companion diagnostics**<br>•Afatinib<br>•Crizotinib<br>•Gefitinib
Medullary Thyroid Cancer	**Target agents against a tumor with a ubiquitous molecular alteration**<br>•Cabozantinib<br>•Vendetanib
Melanoma	**Targeted agents companion diagnostics**<br>•Dabrafenib<br>•Trametinib<br>•Vemurafenib

Pancreatic Cancer	**Targeted agents non companion diagnostics**<br><br>•Erlotinib<br><br>•Everolimus<br><br>•Sunitinib
Renal Cell Carcinoma	**Targeted agents non companion diagnostics**<br><br>•Axitinib<br><br>•Bevacizumab<br><br>•Everolimus<br><br>•Pazopanib<br><br>•Sorafenib<br><br>•Sunitinib<br><br>•Temsirolimus
Soft-tissue Sarcoma	**Targeted agents non companion diagnostics**<br><br>•Pazopanib

Supplementary table 2. **FDA-approved companion diagnostic devices for oncology**

Generic Name (Drug Trade Name)	NDA/BLA	Device Trade Name	PMA	Device Manufacturer
Cetuximab (Erbitux)	BLA 125084	TherascreenKRAS RGQ PCR Kit	P110030	Qiagen Manchester, Ltd.
Cetuximab (Erbitux)<br>Panitumumab (Vectibix)	BLA 125084<br>BLA 125147	Dako EGFR PharmDx Kit	P030044 S001–S002	Dako North America, Inc.
Afatinib (Gilotrif)	NDA 201292	Therascreen EGFR RGQ PCR Kit	P120022	Qiagen Manchester, Ltd.
Imatinib mesylate (Gleevec/Glivec)	NDA 021335<br>NDA 021588	DAKO C-KIT PharmDx	P040011 S001–S002	Dako North America, Inc.
Trastuzumab (Herceptin)	BLA 103792	INFORM HER2 NEU	P940004 S001	Ventana Medical Systems, Inc.
Trastuzumab (Herceptin)	BLA 103792	PATHVYSION HER2 DNA Probe Kit	P980024 S001–S010	Abbott Molecular, Inc.
Trastuzumab (Herceptin)	BLA 103792	PATHWAY ANTI-HER2 NEU (4B5)<br>Rabbit monoclonal primary antibody	P990081 S001–S016	Ventana Medical Systems, Inc.
Trastuzumab (Herceptin)	BLA 103792	INSITE HER2 NEU KIT	P040030	Biogenex Laboratories, Inc.
Trastuzumab (Herceptin)	BLA 103792	SPOT-LIGHT HER2 CISH Kit	P050040 S001–S003	Life Technologies, Inc.
Trastuzumab (Herceptin)	BLA 103792	Bond Oracle Her2 IHC System	P090015	Leica Biosystems
Trastuzumab (Herceptin)	BLA 103792	HER2 CISH PharmDx Kit	P100024 S001–	Dako Denmark A/S

			S004	
Trastuzumab (Herceptin)	BLA 103792	INFORM HER2 DUAL ISH DNA Probe Cocktail Assay	P100027 S001–S006	Ventana Medical Systems, Inc.
Trastuzumab (Herceptin)<br>Pertuzumab (Perjeta)<br>Ado-trastuzumab emtansine (Kadcyla)	BLA 103792<br>BLA 125409	HercepTest	P980018 S001–S017	Dako Denmark A/S
Trastuzumab (Herceptin)<br>Pertuzumab (Perjeta)<br>Ado-trastuzumab emtansine (Kadcyla)	BLA 103792<br>BLA 125409	HER2 FISH PharmDx Kit	P040005 S001–S009	Dako Denmark A/S
Tramatenib (Mekinist)<br>Dabrafenib (Tafinlar)	NDA 204114;<br>NDA 202806	THxID BRAF Kit	P120014	bioMerieux, Inc.
Erlotinib (Tarceva)	NDA 021743	Cobas EGFR Mutation Test	P120019	Roche Molecular Systems, Inc.
Crizotinib (Xalkori)	NDA 202570	VYSIS ALK Break Apart FISH Probe Kit	P110012 S001–S003	Abbott Molecular, Inc.
Vemurafenib (Zelboraf)	NDA 202429	COBAS 4800 BRAF V600 Mutation Test	P110020 S001–S006	Roche Molecular Systems, Inc.
